# Enhanced Effectiveness of Onabotulinum Toxin A in Combination With Definisse KP1 Eye Contour Cream for Upper Face Rejuvenation: A Case Series

**DOI:** 10.1111/jocd.70743

**Published:** 2026-03-16

**Authors:** Ilaria Proietti, Martina Manni

**Affiliations:** ^1^ Department of Medical‐Surgical Sciences and Biotechnologies, Dermatology Unit “Daniele Innocenzi” Sapienza University of Rome Latina Italy; ^2^ RELIFE Srl Florence Italy

**Keywords:** aging, bioactive peptide, collagen, onabotulinum toxin A, SA1‐III (KP1), serpin 1, skin quality, wrinkles

## Abstract

**Background:**

Among antiaging treatments, botulin toxin A injection is one of the most widely used. KP1 (Serpine A1‐III) is a recently developed bioactive peptide that increases collagen type I by enhancing its synthesis and preventing its degradation.

**Aims:**

This case series described the combinatorial effect of onabotulinum toxin A injection with Definisse KP1 eye contour cream application in the treatment of skin aging in the upper third of the face.

**Patients/Methods:**

Adult subjects with moderate to severe dynamic wrinkles who received onabotulinum toxin A combined with Definisse KP1 eye contour cream (Onabotulinum toxin A + KP1 group, hereinafter referred to as + KP1 group) or onabotulinum toxin A alone (Control group) were included in the analysis. The following variables were measured: severity of the disorder, skin hypersensitivity, erythema, oedema, periocular wrinkles, skin quality, hydration‐creepiness, and tightness; each parameter was evaluated in both eyes 4 weeks posttreatment.

**Results:**

Data from 22 in the + KP1 group and 19 in the Control group were collected. In the + KP1 group, all variables improved, except for eye erythema and eye oedema. Conversely, in the Control group, only eye impairment severity and periocular wrinkles improved from baseline. In the satisfaction questionnaire, the overall evaluation of the protocol achieved higher scores in the + KP1 group than in the Control group.

**Conclusions:**

From this case series, the application of Definisse KP1 eye contour cream seems to enhance the effect of onabotulinum toxin A injection on periocular wrinkles and provide additional benefits in skin quality; the combined procedure was tolerated, feasible, and appreciated by subjects.

## 1 Introduction

Aging is the result of a genetically programmed, chronological process, i.e., intrinsic aging, overlying to the outcomes of exogenous insults in the short term or long term, i.e., extrinsic aging [1, 2]; typical signs of an intrinsically aged skin are fine wrinkles, dryness, pale in color, and less elasticity.

Each layer of the skin is involved in the aging processes and shows structural and metabolic changes. The epidermal turnover tends to decline, the epidermis becomes thinner, wound healing gets slower, and desquamation is less effective; the increasing flattening out of the dermal–epidermal junction determines progressive skin fragility and decreased nutrient delivery. Furthermore, the loss in interface area between the epidermis and dermis may induce the separation of these two layers, with potential wrinkles formation. The reduction of dermis thickness occurs along with a reduction in vascularity and cellularity as well as the quantity of fibroblasts and mast cells. The content levels of hyaluronic acid and glycosaminoglycans in this layer decline, and the depletion of collagen and elastin leads to the disorganization of connective tissue and causes the rise of wrinkles. In the hypodermis, site‐specific changes, including atrophy and hypertrophy, occur: the laxity of cutaneous muscles with their aponeuroses increases with age and has a significant impact on facial aging. Facial movements, through contraction of facial muscles, are correlated with facial wrinkles, both dynamic (i.e., occurring along with contraction) and static (i.e., appearing constantly and more noticeable with contraction) [1].

Antiaging treatments have been developed over the past few decades to counteract aging signs and symptoms since skin health is thought to be one of the main indicators reflecting overall well‐being and the perception of health in people [3]. Among these procedures, botulin toxin A injection is one of the most widely used. Botulinum toxin A is a potent neurotoxin that inhibits the release of acetylcholine at the neuromuscular junction, thus favoring localized muscle relaxation when injected in small amounts into specific overactive muscles. The effects of botulinum toxin take roughly 2 weeks to completely manifest and persist for 3–4 months [4]. After the contraindications are carefully evaluated, a personalized amount of botulin toxin A can be injected into the targeted facial muscles by considering the anatomical structure of the face, the targeted area, injection direction and depth, and gender. The application of an appropriate dose of botulin toxin A may erase lines on the forehead and around the eyes, and drooping eyelids [5].

On the other hand, modulating collagen turnover is the basis of another recently proposed antiaging strategy. Serine protease inhibitor (serpin) A1 blocks the activity of the neutrophil elastase, a protease released by phagocytic cells during tissue damage to degrade extracellular matrix components and allow the infiltration of leukocytes and pro‐inflammatory cytokines [6, 7]. The C‐terminal portion of Serpin A1 comprises the putative cleavage site of collagenase and neutrophil elastase; from this region, the bioactive decapeptide SA1‐III (KP1) Ac‐Met‐Gly‐Lys‐Val‐Val‐Asn‐Pro‐Thr‐Gln‐Lys‐NH2 has been identified that demonstrated an impact on collagen production [7]. In vitro, KP1 significantly elicited skin fibroblasts' synthesis of collagen I, increased levels of both soluble collagen in culture media comparable to positive controls, such as L‐ascorbic acid and transforming growth factor‐beta 1, and extracellular collagen, mostly by lowering degradation caused by matrix metallo‐protease‐2 (MMP‐2) and MMP‐9 activity [8]. Furthermore, KP1 can prevent collagen degradation by acting as a competitive substrate for elastase and collagenase, thus resulting in decreased digestion of the target proteins, such as collagen and elastin [7].

KP1 formulae have been made available in face serum, face cream, and eye contour cream that have been tested in and demonstrated their anti‐age efficacy and tolerability. In particular, Definisse KP1 revitalizing eye contour cream containing KP1 improved puffiness, deep and superficial wrinkles, and improved skin dryness around the eye, dark circles, and crow's feet when applied twice daily on 20 healthy female volunteers aged 45–60 years [8].

In this case series, we described the combinatorial effect of onabotulinum toxin A injection with Definisse KP1 revitalizing eye contour cream application in the treatment of skin aging in the upper third of the face, compared to onabotulinum toxin A injection alone.

## 2 Materials and Methods

### 2.1 Study Design

This case series described the effectiveness and safety of onabotulinum toxin A, with or without the addition of Definisse KP1 revitalizing eye contour in the treatment of the upper third of the face in a single‐center in Italy, from January to December 2024.

To be considered for the data collection, subjects should be adults aged 18–65 years, present moderate to severe dynamic wrinkles in the upper third of the face, including the glabellar lines, forehead lines, and crow's feet, have a Fitzpatrick skin type I–IV, have not received any prior treatments with botulinum toxin or other neuromodulators in the last 6 months, and have good overall health without systemic conditions that could interfere with treatment.

Data were collected according to relevant local laws and principles outlined in the Helsinki Declaration.

### 2.2 Procedures

Subjects were treated with onabotulinum toxin A combined with Definisse KP1 revitalizing eye contour cream (+ KP1 group) or onabotulinum toxin A without the addition of any topical cream (Control group).

All botulinum toxin injections were administered by experienced practitioners, following standard injection protocols for the treatment of the upper third of the face. The injection sites included the glabellar lines (vertical lines between the eyebrows caused by corrugator and procerus muscle activity), forehead lines (horizontal lines across the forehead resulting from frontalis muscle contraction), and crow's feet (lateral periorbital lines due to orbicularis oculi muscle activity). Dosages were adjusted based on individual patient needs and anatomical considerations, within the range of 50–64 units.

Definisse KP1 revitalizing eye contour cream is a product designed to improve skin elasticity, hydration, and texture. The cream was applied by subjects at home twice daily—once in the morning and once in the evening—for the entire duration of the study. Subjects were instructed to gently massage a small amount of the cream around the periorbital area, avoiding direct contact with the eyes.

In the presence of one of the following conditions, subjects could not receive the treatment with onabotulinum toxin A or the Definisse KP1 revitalizing eye contour and were not considered for this analysis: (i) known hypersensitivity or allergy to any component of onabotulinum toxin A or the Definisse KP1 eye contour; (ii) previous facial surgery or invasive aesthetic procedures in the upper third of the face within the last year; (iii) dermal filler treatments in the targeted areas within the past 6 months; (iv) presence of any active dermatological condition in the treated area, such as eczema, psoriasis, or active skin infections; (v) excessive skin laxity, severe ptosis, or other anatomical features in the upper face deemed unsuitable for treatment with botulinum toxin by the investigator; (vi) history of neuromuscular disorders, such as myasthenia gravis, Eaton‐Lambert syndrome, or amyotrophic lateral sclerosis; (vii) use of medications that could interfere with neuromuscular function, including aminoglycosides or muscle relaxants, within 2 weeks before treatment; (viii) history of psychological conditions, including body dysmorphic disorder; (ix) pregnant or breastfeeding women.

### 2.3 Imaging Acquisition

To ensure an objective and precise assessment of treatment outcomes, the Vectra H1 (Canfield Scientific, NJ, USA) imaging system was employed for high‐resolution 3D image acquisition. This imaging system is composed of compact 3D cameras that rely on the principle of passive stereophotogrammetry. A single 3D image is created by superimposing three photographs taken from various angles. To standardize the shooting distance, the cameras are equipped with a dual‐beam pointer and a flash system. Before image acquisition, strict positioning guidelines were followed to guarantee uniformity and enable a precise comparison of pre‐ and posttreatment conditions. Images were captured as usual before treatment (baseline), 2 weeks posttreatment, and 4 weeks posttreatment. This advanced imaging technology allowed for detailed evaluation of wrinkle reduction, skin texture improvement, and overall aesthetic outcomes [9].

Additionally, the FaceSculptor software was used to analyze and quantify changes in facial topography. The software enabled precise measurements of wrinkle depth, skin smoothness, and volume changes in the treated areas, providing a comprehensive assessment of treatment efficacy. The combination of Vectra H1 imaging and FaceSculptor ensured consistent, reproducible, and objective data collection across all study participants.

### 2.4 Variables

To describe the reduction in wrinkle severity and improvement in skin quality 4 weeks posttreatment and the subjective patient satisfaction, the following variables were assessed: severity of the disorder, skin hypersensitivity, erythema, oedema, periocular wrinkles, skin quality, hydration‐creepiness, and tightness; each parameter was evaluated in both eyes. A score from 0 to 3 was used to quantitatively define the changes from baseline to the follow up 1 month after the treatment, according to the definitions reported in Table 1. Subjects were asked to answer a series of questions on perceived aesthetic improvement and overall satisfaction with the treatment.

**TABLE 1:** Variable definition.

Variable	Definition	Grade	
Severity of the disorder	The extent or intensity of the disease for which treatment has been applied (wrinkles, PIH/PIE, hyperpigmentation)	0	No sign or symptom of skin disorder
		1	Mild signs or symptoms. The disorder does not cause significant discomfort
		2	Moderate signs or symptoms. The disorder may cause significant discomfort
		3	Severe signs or symptoms. The disorder causes significant discomfort
Skin hypersensitivity	The skin reacts excessively or abnormally to stimuli or substances (it may manifest with symptoms such as redness, itching, swelling, or pain)	0	No hypersensitivity
		1	Mild hypersensitivity
		2	Moderate hypersensitivity
		3	Severe hypersensitivity
Erythema	Redness due to increased blood flow, which may indicate inflammation or irritation	0	No redness
		1	Mild redness
		2	Moderate redness
		3	Severe redness, overt inflammation
Oedema	Swelling caused by fluid accumulation in tissues	0	No edema
		1	Mild edema
		2	Moderate edema
		3	Severe edema
Periocular winkles	Overall evaluation	0	Not noticeable
		1	Slightly noticeable
		2	Moderately noticeable
		3	Extremely noticeable
Skin quality	Firmness, skin evenness, tone evenness, skin glow	0	Excellent overall skin quality
		1	Good overall skin quality
		2	Scarce overall skin quality
		3	Poor overall skin quality
Hydration‐creepiness	Roughness and/or desquamation. Skin that is thin, wrinkled, similar in appearance to crepe paper	0	Highly hydrated, glowing skin
		1	Moderate skin hydration, slight dryness
		2	Reduced skin hydration, evident dryness
		3	Very dry skin, pronounced desquamation
Tightness	The resistance of skin against mechanical force	0	Thigh and compact skin
		1	Moderate skin tightness, slightly relaxed skin
		2	Reduced skin tightness, clearly relaxed skin
		3	Loss of skin tightness, very relaxed skin

All adverse events and their severity throughout the procedures were also collected, and skin texture improvement in the + KP1 group was assessed, focusing on hydration, elasticity, and smoothness as observed through imaging data and patient feedback.

### 2.5 Statistical Analysis

Statistical analysis was performed using SPSS version 21 (SPSS, Chicago, IL, UA) licensed statistical programs. The different variables contained in the study were analyzed with descriptive statistics. Continuous data were expressed as medians with ranges. Categorical variables were expressed as numbers and percentages. For paired sample analysis, the Wilcoxon signed‐rank test was used. Nonparametric Mann–Whitney *U*‐tests were used for the comparison of the different groups (Control group vs. + KP1 group). All *p*‐values were two‐sided, and a *p*‐value < 0.05 was considered statistically significant.

## 3 Results

A total of 41 subjects were considered for this case series, 22 were treated with Onabotulinum toxin A + KP1 (+ KP1 group) and 19 were included in the Control group. Median age was 54 years (min–max 21–82) and most subjects were women with only two men being involved. Overall, Fitzpatrick skin type was II in 6 subjects, III in 23 subjects, and IV in 12 subjects; none had Fitzpatrick skin type I. Table 2 summarizes the baseline characteristics of each group. In all cases, the presence of wrinkles was the reason for treatment.

**TABLE 2:** Baseline characteristics.

	+ KP1 group (*n* = 22)	Control group (*n* = 19)
Age		
Median (min–max)	55 (21–72)	55 (44–82)
Mean (SD)	54.7 (9.7)	57.8 (10.1)
Male, *n*	1	1
Fitzpatrick skin type		
I	0	0
II	3	3
III	14	9
IV	5	7

As reported in Table 3, in the + KP1 group, onabotulinum toxin A injection and Definisse KP1 revitalizing eye contour application significantly improved skin quality from baseline in the following parameters measured: severity of the disorder, skin hypersensitivity, periocular wrinkles, skin quality, hydration‐creepiness, and tightness; eye erythema and eye oedema did not ameliorate. Conversely, in control subjects treated with onabotulinum toxin A, only severity of the disorder and periocular wrinkles significantly improved.

**TABLE 3:** Assessment of wrinkles and skin quality before (T0) and after the treatment (T1).

	+ KP1 group *N* = 22			Control group *N* = 19			T0	T1
	T0	T1	*p* T0 vs T1	T0	T1	*p*	+ KP1 group vs. control group	+ KP1 group vs. control group
	Median (range)	Median (range)		Median (range)	Median (range)			
Severity of the disorder right eye	3 (1–3)	0 (0–0)	< 0.0001	3 (2–3)	1 (0–2)	< 0.0001	0.110	0.0001
Severity of the disorder left eye	3 (1–3)	0 (0–0)	< 0.0001	3 (2–3)	1 (0–2)	< 0.0001	0.507	0.0001
Skin hypersensitivity right eye	1 (0–3)	0 (0–0)	< 0.0001	1 (0–2)	1 (0–2)	0.317	0.422	0.0001
Skin hypersensitivity left eye	1 (0–3)	0 (0–0)	< 0.0001	1 (0–3)	0 (0–2)	0.026	0.568	0.0001
Erythema right eye	0 (0–1)	0 (0–0)	0.025	0 (0–1)	0 (0–1)	0.999	0.119	0.282
Erythema left eye	0 (0–0)	0 (0–0)	0.999	0 (0–0)	0 (0–0)	0.999	1000	1000
Edema right eye	0 (0–3)	0 (0–0)	0.003	0 (0–3)	0 (0–3)	0.102	0.269	0.056
Edema left eye	0 (0–3)	0 (0–0)	0.007	0 (0–2)	0 (0–2)	0.18	0.335	0.056
Periocular wrinkles right eye	3 (2–3)	0 (0–0)	< 0.0001	3 (2–3)	1 (0–3)	< 0.0001	0.643	0.0001
Periocular wrinkles left eye	3 (2–3)	0 (0–0)	< 0.0001	3 (2–3)	1 (0–2)	< 0.0001	0.819	0.0001
Skin quality right eye	3 (2–3)	0 (0–0)	< 0.0001	3 (1–3)	3 (0–3)	0.026	0.452	0.0001
Skin quality left eye	3 (2–3)	0 (0–0)	< 0.0001	3 (1–3)	3 (0–3)	0.026	0.452	0.0001
Hydration‐creepiness right eye	3 (0–3)	0 (0–0)	< 0.0001	2 (0–3)	2 (0–3)	0.102	0.128	0.0001
Hydration‐creepiness left eye	3 (1–3)	0 (0–0)	< 0.0001	3 (0–3)	2 (0–3)	0.059	0.122	0.0001
Tightness right eye	3 (1–3)	0 (0–1)	< 0.0001	2 (1–3)	2 (0–3)	0.01	0.303	0.0001
Tightness left eye	2 (1–3)	0 (0–1)	< 0.0001	2 (1–3)	2 (0–3)	0.005	0.560	0.0001

*Note:* A comparison of scores was performed among baseline to T1 and among the two groups at baseline and T1. Values were considered statistically significant with *p* < 0.05.

At baseline, no differences emerged among the two groups in all parameters considered and all variables became significantly different after the treatment, except for eye erythema and eye oedema. This latter showed a tendency toward an improvement; however, without reaching statistical significance (Table 3).

A subgroup analysis of subjects ≥ 55 years old (*n* = 19) confirmed the results of the overall population. In the + KP1 group (*n* = 12), significant improvements from baseline were observed in all variables, except for erythema; in this subgroup, even eye oedema ameliorated. In the Control group, only periocular wrinkles and the severity of the disorder significantly improved (*n* = 7).

**FIGURE 1:**
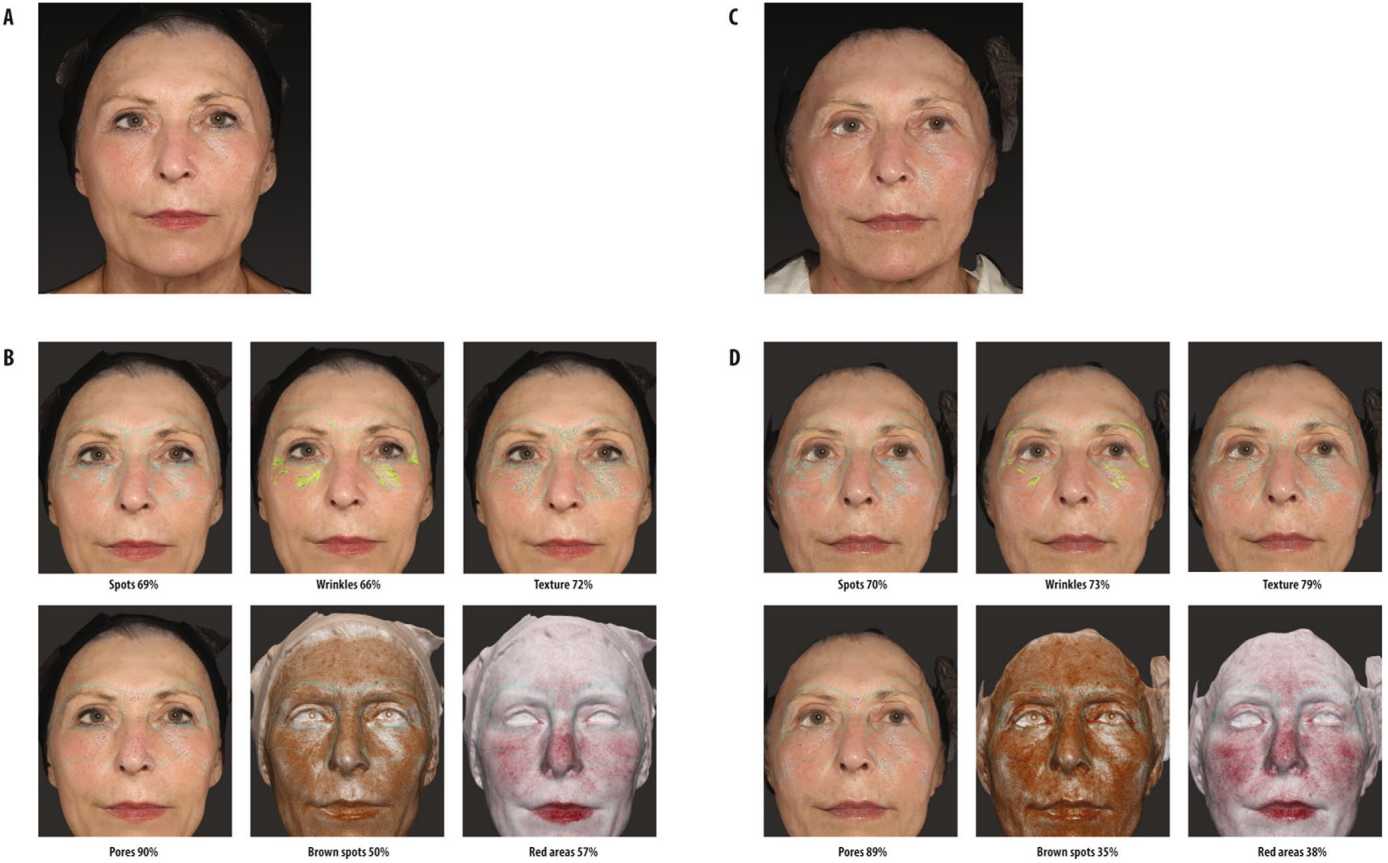
Representative patient's facial appearance before and after treatment with onabotulinum toxin A (Onabotulinum toxin A group). (A) Frontal image at baseline. (B) FaceSculptor analysis of the specified variables at baseline. (C) Frontal image 2 months posttreatment. (D) FaceSculptor analysis of the specified variables 2 months posttreatment.

**FIGURE 2:**
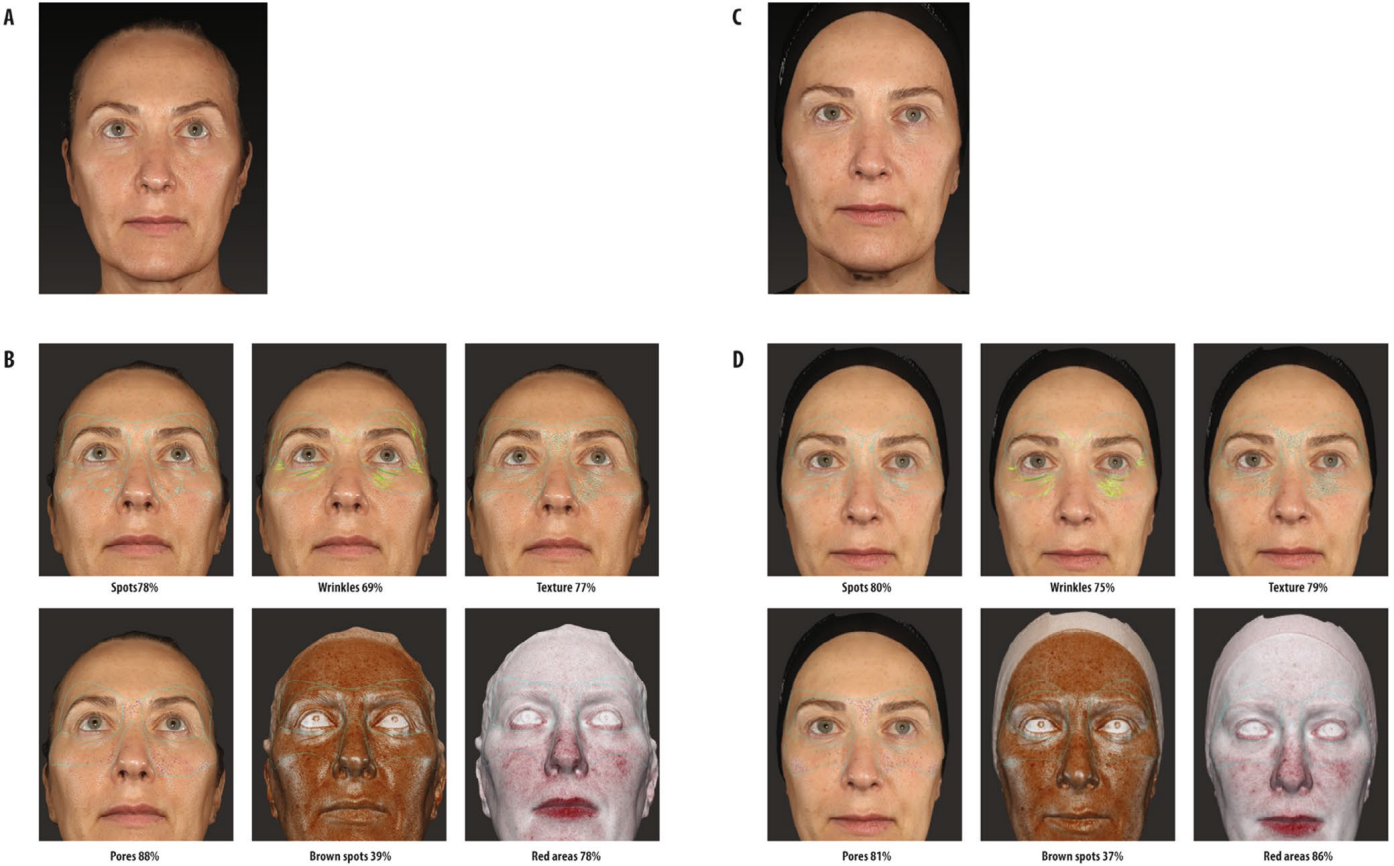
Representative patient's facial appearance before and after treatment with onabotulinum toxin A plus Definisse KP1 eye contour cream (Onabotulinum toxin A + KP1 group). (A) Frontal image at baseline. (B) FaceSculptor analysis of the specified variables at baseline. (C) Frontal image 2 weeks posttreatment. (D) FaceSculptor analysis of the specified variables 2 weeks posttreatment.

Figures 1 and 2 show the facial appearance before and after treatment in two representative subjects, both over 55 years old, from the Control group and + KP1 group, respectively.

Four subjects (two in the + KP1 group and two in the Control group) reported the appearance of hematomas; other adverse events were not recorded.

In both groups, subjects were satisfied with the treatment; however, in the + KP1 group, mean scores were higher than those reported by controls. According to the subjects' feedback, injection of onabotulinum toxin A and application of Definisse KP1 Revitalizing Eye Contour cream improved skin quality, was tolerable, and effective in ameliorating cutaneous disorder, more than onabotulinum injection alone; the overall evaluation of the protocol achieved higher scores in + KP1 group than in Control group (Table 4).

**TABLE 4:** Subjects' satisfaction with the treatment (0 = not at all; 10 = highest value).

	+ KP1 group	Control group
Do you think the treatment protocol improves the appearance of the skin?	9.8	8.9
Do you think the treatment protocol is well tolerated by the skin?	10	8.6
Do you think the treatment protocol is the ideal complement to support skin recovery and post‐onabotulinum toxin A results?	9.8	8.3
Do you think the treatment protocol improves the skin disorder?	9.6	8.5
What is your overall opinion of the treatment protocol?	10	8.6

## 4 Discussion

Data collected showed that a concomitant injection of onabotulinum toxin A and application of Definisse KP1 Revitalizing Eye Contour cream improved skin quality and wrinkle severity compared to onabotulinum toxin A injection alone. The treatment was well tolerated and appreciated by subjects who gave higher rating scores to the combination.

The injection of onabotulinum toxin A in the aesthetic field requires careful initial assessment of the subject in its complexity and individuality [10]. Its use is consolidated, and several years of experience result in sophisticated treatment approaches, more specific targeted injections, and provide a better understanding of facial aging, leading to satisfying therapeutic results for both subjects and clinicians [11].

KP1 is a recently developed bioactive peptide that increases collagen type I by enhancing its synthesis and preventing its degradation [7]. Previous studies with KP1, reviewed by Rovero et al., indicated that formulations containing KP1 improved skin hydration, elasticity, and density. Definisse revitalizing eye contour cream improved dark circles (−10.7% change in bluish or brown pigmentation by colorimetry) and crow's feet (−19.2% change in average roughness by profilometry) (both *p* < 0.05 vs. baseline) [8].

Based on our knowledge, this procedure is the first where two mechanisms of action were added to ameliorate skin quality and provide deeper antiaging effects. Previous reports investigated the application of topical creams (eutectic lidocaine/prilocaine cream—EMLA) for pain relief during multiple botulinum toxin type A [12] and periocular injection [13]. A recent case series described the real‐life experiences of a topical neuropeptide serum containing 2% acetyl hexapeptide‐8, 2% dipeptide diaminobutyroyl, 5% polyhydroxy acids (PHA), 5% niacinamide, and 1% laminaria extract (topical neuropeptide serum [TNP‐serum]) that was applied in combination with botulinum A injections in five subjects; the TPN‐serum ameliorated wrinkles, skin radiance, redness, and pore sizes. The mechanism of action of TPN‐serum is based on enhancing collagen type I, III, and IV and elastin expression [14]. Although limited to an anecdotal series of subjects, these observations may support our results that magnify the effectiveness of botulinum injection on wrinkles by adding an effect on skin quality.

One strength of our study was to quantify the changes of skin variables from baseline by an instrumental analysis performed with Vectra H1 imaging and FaceSculptor. The Vectra H1 imaging system with Visia camera is currently viewed as the gold‐standard system for visualizing the face and providing objective skin analyses. Previous studies independently examined the system and the measurement methods of skin aspects, deeming it a useful tool for wrinkle assessment [15]. The results that emerged from the FaceSculptor analysis, performed for each participant, provided an objective comparison of treatment effects. Furthermore, the lack of improvement in eye erythema and eye oedema could be considered an internal control that supports the specific action of Definisse KP1 Revitalizing Eye Contour cream combined with onabotulinum toxin A injection on wrinkles and skin wellbeing.

Lastly, given the importance of the patient's perception of his/her wellbeing during and after the treatment, it is important to highlight the satisfaction with the Definisse KP1 revitalizing eye contour cream application that most subjects considered as an ideal complement to onabotulinum toxin A injection. The overall protocol was positively judged, thus supporting the feasibility in clinical practice.

The main limitation of the case series was the limited population size that could, however, provide initial interesting insights on the potentiality of integration of KP1‐based cream in aesthetic procedures.

In conclusion, the application of Definisse KP1 revitalizing eye contour cream enhanced the effect of onabotulinum toxin A injection on periocular wrinkles and provided additional benefits in skin quality, as clearly shown in the change of score from 3 to 0 for severity of the disorder, skin hypersensitivity, periocular wrinkles, skin quality, hydration‐creepiness, and tightness. The combined procedure was tolerated, feasible, and appreciated by subjects. This strategy is promising to manage periocular wrinkles and favor skin wellbeing of a wide range of subjects: indeed, the procedure was safe and feasible in adult subjects of all ages and with Fitzpatrick skin type from II to IV. No data were available on Fitzpatrick skin type I, as this phenotype is less frequent in Italy.

## Author Contributions

I.P. contributed to the project by conducting research, collecting data, drafting the manuscript, and revising the final submitted version.

## Ethics Statement

The study adhered to all relevant local laws and principles outlined in the Helsinki Declaration. Written informed consent was obtained from the patient, including consent for the publication of clinical information and images.

## Conflicts of Interest

I.P. has no conflicts of interest to declare; M.M. is employed at Relife Srl.

## Data Availability

The data that support the findings of this study are available from the corresponding author upon reasonable request.
